# QTL mapping of selenium content using a RIL population in wheat

**DOI:** 10.1371/journal.pone.0184351

**Published:** 2017-09-07

**Authors:** Pei Wang, Huinan Wang, Qing Liu, Xia Tian, Yanxi Shi, Xiaocun Zhang

**Affiliations:** 1 College of Food Science and Engineering, Shandong Agricultural University, Tai’an, Shandong Province, China P.R.; 2 State Key Laboratory of Crop Biology, Shandong Key Laboratory of Crop Biology, College of Agronomy, Shandong Agricultural University, Tai’an, Shandong Province, China P.R.; 3 College of Resources and Environment, Qingdao Agricultural University, Chengyang, Qingdao, Shandong Province, China P.R.; Institute of Genetics and Developmental Biology Chinese Academy of Sciences, CHINA

## Abstract

Selenium (Se) is an essential trace element that plays various roles in human health. Understanding the genetic control of Se content and quantitative trait loci (QTL) mapping provide a basis for Se biofortification of wheat to enhance grain Se content. In the present study, a set of recombinant inbred lines (RILs) derived from two Chinese winter wheat varieties (Tainong18 and Linmai6) was used to detect QTLs for Se content in hydroponic and field trials. In total, 16 QTLs for six Se content-related traits were detected on eight chromosomes, 1B, 2B, 4B, 5A, 5B, 5D, 6A, and 7D. Of these, seven QTLs were detected at the seedling stage and nine at the adult stage. The contribution of each QTL to Se content ranged from 7.37% to 20.22%. *QSsece-7D*.*2*, located between marker loci D-3033829 and D-1668160, had the highest contribution (20.22%). This study helps in understanding the genetic basis for Se contents and will provide a basis for gene mapping of Se content in wheat.

## Introduction

Selenium (Se) is an essential trace element that exhibits tumor-inhibiting, immunity-enhancing, and cancer-preventing actions in humans [1, 2]. Se deficiency usually results in serious health damage [3, 4]. The World Health Organization (WHO) recommends a daily intake of 50–200 μg Se for optimum human health [5]. However, according to surveys conducted by Golubkina and Blazina [6, 7], Se intake is low in various countries, and about 0.5–1 billion people globally may have insufficient Se intake [8].

Biofortification is the most economical and sustainable strategy for alleviating Se deficiency [9, 10]. Wheat (*Triticum aestivum* L., 2n = 6x = 42, AABBDD) is one of the most important food crops in the world, and its derivative products, such as breads, cakes, and pasta are important sources of Se in the human diet [6, 11, 12]. Thus, improving Se uptake in wheat populations by increasing the Se content would be advantageous [9].

As physiological traits related to Se uptake and accumulation are genetically controlled, a detailed knowledge of the genetics is important for Se biofortification. Traits associated with Se uptake are typically quantitative. Quantitative trait locus (QTL) analysis provides an effective approach to dissect a complex, quantitative trait into component loci to study their relative effects on the trait [13]. Recently, QTL analyses of Se content in lentils [14] and rice [15, 16] have been reported. Pu et al. [17] detected five QTLs controlling Se concentration in wheat, on chromosomes 3D, 4A, 4D, 5B, and 7D, using two recombinant inbred lines (RILs). However, QTL mapping studies of Se uptake traits of wheat are rare. Therefore, it is important to dissect the genetic basis of Se uptake traits using more genetic populations.

In this study, we used a set of RILs derived from two Chinese winter wheat varieties (Tainong18 [TN18] and Linmai6 [LM6]) to detect QTLs for Se content in plants at the seedling and adult stages.

## Materials and methods

### Plant materials

The population for QTL analysis was a set of RILs derived from “TN18 × LM6” (TL-RIL, F_8_ in 2013) by single-seed descent. TN18 is a cultivar developed by our group that was released in 2008 and is planted on approximately 300,000 ha/year in the Huang-huai winter wheat region of China. TN18 possesses several salient features, such as high grain yield and fine quality. The male parent, LM6, is an elite breeding line developed by the Linyi Academy of Agricultural Sciences, China. In total, 184 RILs were randomly selected from the original 305 lines and were used for constructing the genetic map and for phenotypic investigations.

### Experimental design and implementation

#### Hydroponic culture trial

The 184 RILs and their parents were grown under hydroponic culture in a greenhouse at Shandong Agricultural University in 2014 and 2015. Hoagland’s nutrient solution [18] amended for wheat growth (Table 1) was used. Two Se treatments (0 and 0.1 μmol/L Na_2_SeO_3_) were once applied in this experiment. A randomized block design with three replications was employed, with the replications as the main plots, and subplots for the genotypes. Fifty seeds of each line and their parents were sterilized for 5 min in 10% H_2_O_2_, washed with distilled water, and germinated on moist filter paper in Petri dishes for 7 days. We selected three uniform seedlings for each line of each replication, with both an embryogenic primary root and a coleoptile of 3–4 cm long, and transferred them to perforated trays placed on plastic tanks containing 20 L of nutrient solution. Containers and tops for hydroponic culture were opaque to produce healthy roots and discourage algal growth. The planting distance was 3 cm (within as well as between rows). The nutrient solution was continuously aerated through rubber tubes connected to an air compressor and was renewed every four days. The plants were grown for 30 days (from March 13, 2014 to April 13, 2014 and from March 15, 2015 to April 15, 2015). The temperature, relative humidity, and photoperiod were measured and recorded every 10 min using data loggers (ZDR, Zhejiang University Electric Equipment Factory, China).

**Table 1:** Nutrient solution composition for wheat seedling growth.

Element	Concentration mmol/L	Element	Concentration μmol/L
KH_2_PO_4_	0.2	H_3_BO_3_	1
MgSO_4_·7H_2_O	0.5	(NH_4_)_6_Mo_7_O_24_·4H_2_O	0.1
KCl	1.5	CuSO_4_·5H_2_O	0.5
CaCl_2_	1.5	ZnSO_4_·7H_2_O	1
(NH4)_2_SO4·H_2_O	1	MnSO_4_·H_2_O	1
Ca(NO_3_)_2_·4H_2_O	1	FeEDTA	100

#### Field trials

A 1.5-m^2^ plot with four 1.5 m-long rows, 0.25 m apart, was used. Thirty seeds of each RIL were sown per row on October 1, 2013 and October 4, 2014, and plants were harvested mid-June the following year. The Se content of the top soil (20 cm) was 0.31 mg/kg, and Se solution was sprayed at 76 g/ha Se at the flowering stage in 2014 and 2015. There were no serious diseases during the year 2014 and 2015 in the field, and the diseases were well controlled in our experiment. The expreriments were implemented in Agoronomy Experimental Station, Shandong Agricultural University.

#### Trait measurements

In hydroponic culture, three plants of each line were harvested. The plants were washed with deionized water and excess water was removed using absorbent paper. The roots and shoots were separated with scissors and dried at 105°C for 30 min and then at 75°C for 72 h. Root and shoot dry weights were measured using 1/1,000 balances. In field trials, after harvest, grains of each RIL were mixed thoroughly, rinsed with distilled water, oven-dried, and milled on a 0.5-mm sieve. Dry weight was determined. Se concentration in all samples was analyzed by the standard method of the Code of China (GB 5009.93–2010). Table 2 summarizes the six traits investigated and the methods used to do so.

**Table 2:** Summary of investigated traits and their measurement methods.

Experiment	Trait investigated			
	Trait		Unit	Method for trait measurement
Hydroponic culture trial	RSeCe	Root Se concentration	mg/kg	GB 5009.93–2010
	SSeCe	Shoot Se concentration	mg/kg	GB 5009.93–2010
	RSeC	Root Se content per plant	mg/plant	RSeCe × root dry weight
	SSeC	Shoot Se content per plant	mg/plant	SSeCe × shoot dry weight
Field trial	GSeCe	Grain Se concentration	mg/kg	GB 5009.93–2010
	GSeC	Grain Se content per plant	mg/plant	GSeCe × GWP

### Data analysis

Data were analyzed by analysis of variance (ANOVA) using SAS software (SAS Institute, Cary, NC, USA). The least significant difference (LSD) test and broad-sense heritability (h_B_^2^) assay for the traits were conducted as reported by Knapp et al. [19]. The equation used to calculate heritability was *h*_*B*_^2^ = *σ*_*g*_^*2*^/(*σ*_*g*_^*2*^+*σ*_*e*_^*2*^), where *σ*_*g*_^*2*^ is the genotypic variance, and *σ*_*e*_^*2*^ is the total error variance.

The high-density genetic map for 184 RILs of “TN18 × LM6” [20] was employed in the QTL analysis. The map consists of 10,739 markers assigned to 21 chromosomes, covering a total map length of 3,394.47 cM with a marker density of 0.63 cM. In this map, 5,399 loci are unique loci, including 3,788 DArT, 1,506 SNP and 105 SSR loci, the other 5,340 loci co-segregate with other markers. The Windows QTL Cartographer 2.5 software [21] was used for QTL mapping, and composite interval mapping was selected to search for QTLs of each trait separately for each of the three treatments in hydroponics and field trial. The parameter setup “model 6 standard analysis” was used with a walk speed of 1 cM, “forward and backward” regression for the selection of the markers was used to control for the genetic background, with up to five control markers, and a blocked window size of 10 cM was used to exclude closely linked control markers at the tested site. The LOD threshold for QTL declaration used was that provided by the Windows QTL Cartographer 2.5 software. The LOD threshold for QTL declaration for each trait–treatment combination was defined by 1,000 permutations at *p* ≤ 0.05 [22], and a minimum LOD score of 3.0 was chosen.

## Results

### Phenotypic variation, correlations, and performance

The two parents, TN18 and LM6, showed significant differences for all traits, RSeCe, SSeCe, SSeC, GSeCe, GSeC, and RSeC (Table 3). Similarly, for the RILs, the ANOVA results showed significant differences for all of the investigated traits (*p* ≤ 0.01) (Table 3). The phenotypic values for the traits varied widely among the 184 RILs (Table 4). The coefficients of variation (CVs) ranged from 23.26% (RSeCe, AV) to 92.97% (GSeC, E1), indicating large phenotypic variation between the RILs. The heritability values (*h*_*B*_^*2*^) for the investigated traits ranged from 53.55% (GSeC) to 95.32% (SSeCe). Of these, the h_B_^2^ values for RSeCe, SSeCe, and SSeC were over 80%.

**Table 3:** ANOVA results of the investigated traits.

Traits	Source of variation
RseCe	129.94**
SSeCe	67.88**
RSeC	7.29**
SSeC	25.76**
GSeCe	7.08**
GSeC	4.65**

The abbreviations of traits are defined in Table 2;

**p< 0.01

**Table 4:** Summary statistics of the phenotypic performance for Se content for the RILs and their parents.

Trait	Environment	Parent		RIL population					Heritability
		TN18	LM6	Average	SD	Max	Min	CV (%)	(*h*_*B*_^*2*^)
RSeCe	E1	15.51	11.33	10.24	2.39	16.58	4.36	23.35	81.95
	E2	15.84	11.91	10.09	2.37	15.70	4.09	23.48	
	AV	16.68	11.62	10.14	3.36	15.41	4.23	23.26	
SSeCe	E1	1.44	1.04	0.72	0.30	1.94	0.02	41.84	95.32
	E2	1.51	1.14	0.79	0.35	2.42	0.13	44.65	
	AV	1.48	1.09	0.76	0.32	2.16	0.17	42.31	
RSeC	E1	0.29	0.22	0.21	0.07	0.40	0.01	33.02	79.55
	E2	0.28	0.26	0.15	0.06	0.33	0.04	41.70	
	AV	0.28	0.24	0.18	0.06	0.35	0.05	34.20	
SSeC	E1	0.10	0.06	0.05	0.03	0.18	0.01	50.51	90.00
	E2	0.09	0.06	0.06	0.04	0.22	0.01	60.03	
	AV	0.10	0.06	0.06	0.03	0.20	0.01	53.88	
GSeCe	E1	1.04	0.87	0.70	0.59	3.24	0.08	83.92	68.64
	E2	1.40	0.70	0.57	0.39	2.14	0.01	68.19	
	AV	1.22	0.78	0.64	0.42	2.54	0.07	66.14	
GSeC	E1	0.06	0.05	0.07	0.07	0.38	0.00	92.97	53.33
	E2	0.13	0.05	0.06	0.04	0.23	0.00	74.40	
	AV	0.10	0.05	0.07	0.05	0.25	0.01	68.81	

The abbreviations of traits are defined in Table 2; CV (%) = SD/average × 100%; TN18, Tainong18; LM6, Linmai6; E1, 2014; E2, 2015; AV, average value of 2014 and 2015.

### QTL analyses

**Fig 1:**
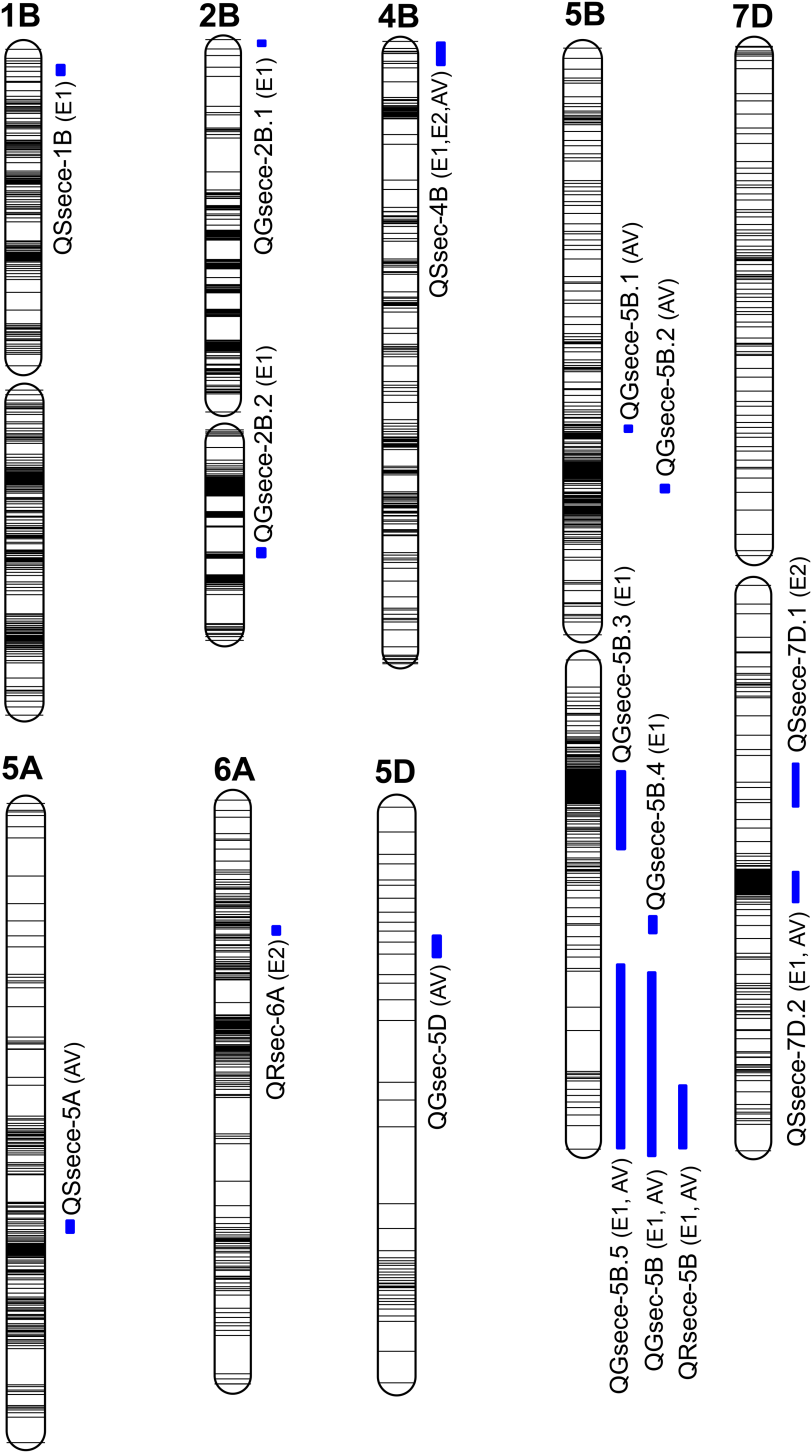
Chromosomal locations of 16 QTLs for the investigated traits based on RILs derived from the cross “TN18 × LM6”. (A) QTLs are indicated on the right side of each chromosome. LOD threshold was set at 3.0. (B) QRsece, QSsece, QRsec, QSsec, QGsece, and QGsec are QTLs for root Se concentration, shoot Se concentration, root Se content per plant, shoot Se content per plant, grain Se concentration, and grain Se content per plant, respectively.

In total, 16 QTLs for six traits related to Se content were detected on eight chromosomes. Among them, seven were QTLs for four seedling traits and nine were QTLs for two adult traits. One QTL for RSeCe (*QRsece-5B)*, with additive effects, was mapped to chromosome 5B (Table 5; Fig 1), and explained 8.91% to 9.58% of the phenotypic variance in two environments (E1 and AV). The additive effect of the QTL for RSeCe was negative, with TN18 increasing the effect of the QTL.

**Table 5:** Additive QTLs for Se efficiency traits at seedling stage under different Se treatments in hydroponic culture.

Trait	QTL	Environment	Marker interval	LOD	Contribution(%)	Additive effect
RSeCe	*QRsece-5B*	AV	gwm67—swes100	4.10	8.91	–0.73
		E1	wPt-5120—gwm67	4.21	9.58	–0.78
SSeCe	*QSsece -1B*	E1	wPt-8240—D-4008980	3.88	12.26	0.11
	*QSsece-5A*	AV	D-2264517—D-1010717	3.74	7.71	–0.10
	*QSsece-7D*.*1*	E2	D-1228261—D-1668160	4.54	13.53	–0.13
	*QSsece-7D*.*2*	AV	D-3033829—D-1668160	7.13	20.22	–0.15
		E1	D-1379418—S-2264900	6.53	13.90	–0.11
RSeC	*QRsec-6A*	E2	S-1018959—D-1151780	4.76	10.99	–0.03
SSeC	*QSsec-4B*	E1	wPt-8555—Jagger-c10704-106	5.58	12.48	0.01
		E2	wPt-744595—wPt-7233	4.82	9.70	0.01
		AV	wPt-744595—wPt-7233	3.74	7.37	0.01

Positive additive effect increased effect contributed by LM6; negative effect was contributed by TN18; E1, 2014; E2, 2015; AV, average value of 2014 and 2015.

Four QTLs on three chromosomes (1B, 5A, and 7D) linked to SSeCe explained 7.71% to 20.22% of phenotypic variance, especially, the contributions of *QSsece-1B*, *QSsece-7D*.*1*, and *QSsece-7D*.*2* were high (12.26–20.22%) (Table 5; Fig 1). *QSsece-7D*.*2* was detected in both the E1 and the AV environment. *QSsece-1B* had positive effects contributed by LM6. The other four QTLs (*QSsece-5A*, *QSsece-7D*.*1*, *and QSsece-7D*.*2*) had negative effects and originated from TN18. For RSeC, one QTL, *QRsec-6A*, was detected (Table 5; Fig 1). The phenotypic variance was 10.99%. The additive effect of *QRsec-6A* was negative, with TN18 increasing the effect. One QTL for SSeC was mapped to chromosome 4B (Table 5; Fig 1) and accounted for 7.37–12.48% of the phenotypic variance. *QSsec-4B* was simultaneously detected in three environments (E1, E2, and AV). The additive effect of *QSsec-4B* was positive, and was derived from LM6. Seven QTLs associated with GSeCe were mapped to chromosomes 2B and 5B, among which *QGsece-5B*.*4* accounted for 15.57%, with positive effects from LM6 (Table 6; Fig 1). Six QTLs (*QGsece-2B*.*1*, *QGsece-2B*.*2*, *QGsece-5B*.*1*, *QGsece-5B*.*2*, *QGsece-5B*.*3*, and *QGsece-5B*.*5*) accounted for 7.44–14.36% of GSeCe, with negative effects from TN18. *QGsece-5B*.*5* was simultaneously detected in E1 and AV.

**Table 6:** Additive QTLs for Se efficiency traits at harvest stage under different Se treatments.

Trait	QTL	Environment	Marker interval	LODs	Contribution (%)	Additive effect
GSeCe	*QGsece-2B*.*1*	E1	D-3956657—S-1105975	3.69	12.56	–0.21
	*QGsece-2B*.*2*	E1	S-1120640—D-3024250	4.25	10.42	–0.19
	*QGsece-5B*.*1*	AV	D-3953407—D-1071681	4.57	8.96	–0.17
	*QGsece-5B*.*2*	AV	D-2289135—D-1236560	3.77	7.44	–0.14
	*QGsece-5B*.*3*	E1	RAC875-c33387_888—D-3936732	7.01	13.93	–0.24
	*QGsece-5B*.*4*	E1	S-3064451—D-1238798	4.55	15.57	0.24
	*QGsece-5B*.*5*	E1	D-3022447—S-1020653	4.30	11.75	–0.21
		AV	BS00082312-51—D-1236561	6.68	14.36	–0.21
GSeC	*QGsec-5B*	E1	BS00082312-51—wPt-5120	6.95	14.48	–0.03
		AV	gwm67—swes100	7.96	17.70	–0.02
	*QGsec-5D*	AV	S-3958480—S-2347952	4.36	8.94	0.02

Positive additive effect increased effect contributed by LM6; negative effect was contributed by TN18; E1, 2014; E2, 2015; AV, average value of 2014 and 2015.

Two QTLs for GSeC were mapped to different chromosomes (5B and 5D) (Table 6; Fig 1). *QGsec-5B* was contributed by TN18 and explained 14.48–17.70% of the phenotypic variance. The QTL *QGsec-5D* accounted for 8.94% of GSeC, with TN18 increasing the effect.

## Discussion

### The accumulation of Se in the seedling and grain of wheat

Wheat has a strong ability to accumulate Se. Se concentrations in wheat can reach 196 mg/kg in the roots [12], 387 mg/kg in the shoot [12], and 5.53 mg/kg in the grains [23] in Se-rich regions or upon addition of Se to the soil. In this study, grain Se content reached 3.24 mg/kg (S1 Table). Thus, wheat, as one of the most important human food sources, can indeed play an important role in Se supplementation in humans. Significant genotypic differences in Se content among wheat varieties had also be reported [24–27] and we also detected large variance among the RIL populations. Therefore, it was hopefulness to select and breed Se enriched wheat varieties.

### Important chromosome for Se related traits in wheat grain

Se content in wheat is controlled by genetic and environmental factors. However, few studies have focused on QTLs for Se content. Using two wheat RILs, Pu et al. [17] had detected five QTLs controlling Se concentration, on chromosomes 3D, 4A, 4D, 5B, and 7D, that accounted for 6.4–35.1% of phenotypic variation. In this study, we detected nine QTLs for Se content on three chromosomes (2B, 5Band 5D) and most of the QTLs were firstly identified. Both investigations detected QTLs for Se content of grain on chromosome 5B. Pu et al. [17] detected one QTL for Se concent on chromosome 5B, which explained 10.1% of grain Se content variation. Six out of nine QTLs for Se related traits of grain including five QTLs for GSeCe, one QTL for GSeC were also detected on this chromosome in this study. The phenotypic variance explained by these QTLs ranged from 7.44% to 17.70%, and the contribution of *QGsec-5B* (AV) was the highest (17.70%). In short, chromosome 5B is important for Se concentration and deserves further concern.

### QTLs for Se related traits in seedling and grain

Seedling was an important stage to investigate the absorption and accumulation characteristic of Se in wheat. The results in nutrient solution culture at seedling stage were more precisely due to the relatively stable and precisely controlled environments. We detected one environmental stable QTL QSSeC which can be identified in two different environments. This means this QTL may be an important stable QTL for Se content at seedling stage.

Except for *QRsece-5B*, most QTLs detected for seedling were not consistent with that in grain according to the results of this study. But *QRsece-5B* was located at the same site with *QGsece-5B*.*5* and *QGsec-5B* on 5B. Therefore, there might be a QTL/gene which could simultaneously affect Se concentration of root in seedling and Se content in grain. 7D chromosome might related with Se-related traits of seedling and grain simultaneously. One QTL located on 7D contributed 28.5% to grain Se concent in the study of Pu et al. [17], and we also located two QTLs for SSeCe at seedling stage on 7D. So, chromosome 7D might be related with grain Se and seedling Se content.

### A valuable QTL related with Se content in seedling

We detected a stable QTL (*QSsec-4B*) related with Se content in seedling, which stably expressed in all the environments (E1, E2 and AV). It explained the phenotypic variance from 7.37% to 12.48%. Three DArT markers (wPt-8555, wPt-744595 and wPt-7233) and one SNP marker (Jagger-c10704-106) were associated with this QTL. According the sequence of the four markers, we blasted their alignments by http://www.sequenceserver.com/ and found four scaffolds on 4B chromosome. All the information related with this QTL above were listed in S2, S3 and S4 Tables. These information could be used to further investigate and utilization of this QTL. As we all know, KASP or CAPS/dCAPS markers can be conveniently used by breeders in biofortification, and DArT and SNP markers can be transformed to these type of markers. This QTL only related with seedling Se content and may be used to increase the seedling Se content in the future. As we all know, QTL was only the rough location of the possible genes and the location results were significantly affected by complicated environments. Therefore, the fine mapping of this QTL also need for future research.

## Conclusions

Se properties of wheat in seeding and grain were quantitative traits and each trait was controlled by several different QTLs/genes. Seven and nine QTLs were detected respectively for Se content in seedling and grain of wheat in this investigation. Most of these QTLs related to Se properties of wheat were first be reported and seven out of the total 16 QTLs were located on 5B chromosome. The contribution of these QTLs on 5B ranged from 7.44% to 17.70% and six out of these seven QTLs were related to Se properties of grain. Therefore, the QTLs on 5B for Se properties needs further investigation for their application in Se related gene detection and breeding programs for biofortification toward high-selenium wheat cultivars to improve health.

## Supporting information

S1 Table Se concentration of wheat in hydroponic and field. (PDF)

S2 Table Basic information of the four markers related with *QSsec-4B* on 4B chromosome. (PDF)

S3 Table Sequence of the four markers related with *QSsec-4B* on 4B chromosome. (PDF)

S4 Table Significant alignments of the four markers related with *QSsec-4B* on 4B chromosome. (PDF)
