# Supplementary material for: QTL mapping of selenium content using a RIL population in wheat
Source: PLoS One. 2017 Sep 7;12(9):e0184351. doi: 10.1371/journal.pone.0184351 (PMC5589217; doi:10.1371/journal.pone.0184351)
Supplement: S1 Table — (PDF) [file pone.0184351.s001.pdf]

S1 Table. Se concentration of wheat in hydroponic and field

No.	Hydroponic						Field		
	Root Se concentration (mg/kg)			Shoot Se concentration(mg/kg)			Grain Se concentration（mg/kg)		
	QRsece-E1	QRsece-E2	QRsece-AV	QSsece-E1	QSsece-E2	QSsece-AV	QGsece-E1	QGsece-E2	QGsece-AV
1	8.93	7.21	8.07	0.86	1.06	0.96	0.12	0.65	0.38
2	10.91	10.19	10.55	1.31	1.56	1.44	0.16	0.73	0.45
3	11.51	10.98	11.24	1.01	1.36	1.19	0.25	1.61	0.93
4	11.11	12.01	11.56	1.04	1.14	1.09	0.12	0.61	0.36
5	10.99	11.43	11.21	1.90	2.42	2.16	0.16	1.46	0.81
6	11.11	10.00	10.56	1.01	1.34	1.18	0.23	0.48	0.35
7	10.81	11.35	11.08	1.08	1.51	1.30	0.19	0.51	0.35
8	11.11	11.80	11.45	1.21	1.32	1.26	0.20	0.67	0.44
9	10.80	10.00	10.40	1.44	1.11	1.28	0.29	0.66	0.47
10	11.14	11.00	11.07	1.27	1.08	1.17	0.15	0.39	0.27
11	10.06	10.23	10.15	1.28	1.83	1.56	0.18	0.85	0.51
12	11.92	12.19	12.05	1.16	1.48	1.32	0.21	0.70	0.46
13	12.37	13.64	13.00	1.15	1.41	1.28	0.21	0.73	0.47
14	10.49	11.43	10.96	0.86	1.07	0.97	0.37	0.61	0.49
15	11.67	11.94	11.80	0.53	0.73	0.63	0.76	0.53	0.65
16	10.51	10.17	10.34	0.83	0.93	0.88	0.47	0.29	0.38
17	10.53	10.08	10.31	0.55	0.52	0.53	0.67	0.92	0.80
18	10.05	9.93	9.99	0.69	0.56	0.62	0.36	0.68	0.52
19	11.40	10.60	11.00	0.64	0.55	0.59	1.18	0.92	1.05
20	8.71	9.06	8.89	0.78	0.82	0.80	0.91	0.82	0.87
21	9.23	10.88	10.06	0.67	0.82	0.74	0.56	0.90	0.73
22	9.00	8.68	8.84	0.82	0.93	0.87	0.37	0.05	0.21
23	10.93	11.51	11.22	0.90	0.93	0.92	0.52	0.21	0.37
24	12.49	10.35	11.42	0.51	0.55	0.53	0.60	0.39	0.49
25	10.20	10.03	10.11	0.62	0.63	0.62	2.23	0.18	1.21
26	9.48	9.48	9.48	0.51	0.55	0.53	1.62	0.08	0.85
27	10.08	9.51	9.79	0.49	0.54	0.52	0.40	0.52	0.46
28	9.22	9.47	9.34	0.64	0.78	0.71	0.51	0.64	0.57
29	10.02	9.97	10.00	0.61	0.67	0.64	0.83	0.76	0.80
30	4.36	4.95	4.65	0.71	0.77	0.74	2.04	0.19	1.11
31	7.48	6.06	6.77	0.63	0.77	0.70	0.50	0.51	0.51
32	5.96	4.84	5.40	0.36	0.36	0.36	0.42	0.83	0.63
33	7.15	7.86	7.51	0.66	0.69	0.68	0.40	0.73	0.57
34	8.73	8.93	8.83	0.84	0.87	0.85	2.41	2.04	2.22

35	9.17	9.34	9.25	0.67	0.75	0.71	1.24	0.81	1.02
36	8.96	8.06	8.51	0.45	0.39	0.42	1.84	0.52	1.18
37	7.59	7.13	7.36	0.69	0.66	0.68	0.50	1.55	1.03
38	10.07	9.68	9.88	0.61	0.56	0.59	0.41	0.64	0.53
39	9.84	8.73	9.29	0.02	.	.	0.59	0.01	0.30
40	7.98	8.03	8.01	1.08	0.89	0.98	0.52	0.40	0.46
41	7.98	8.42	8.20	0.59	0.57	0.58	0.64	0.18	0.41
42	9.70	9.09	9.39	0.40	0.44	0.42	0.55	0.41	0.48
43	10.88	9.72	10.30	0.25	0.34	0.30	0.37	0.24	0.30
44	.	11.81	.	0.32	0.35	0.34	0.52	0.40	0.46
45	8.03	8.15	8.09	0.57	0.65	0.61	0.43	.	.
46	9.33	9.09	9.21	0.61	0.66	0.64	0.54	0.43	0.49
47	10.62	11.10	10.86	0.53	0.65	0.59	0.65	0.53	0.59
48	9.50	9.56	9.53	0.65	0.74	0.69	0.60	0.60	0.60
49	8.42	8.26	8.34	0.54	0.55	0.54	0.68	0.29	0.49
50	8.61	9.03	8.82	0.43	0.56	0.50	0.51	0.60	0.55
51	7.03	7.00	7.01	0.79	0.86	0.82	0.52	0.06	0.29
52	10.52	9.86	10.19	0.42	0.49	0.45	1.55	1.16	1.36
53	9.27	8.85	9.06	0.33	0.43	0.38	2.65	1.90	2.28
54	9.78	.	.	0.62	0.81	0.71	0.64	0.60	0.62
55	9.55	9.09	9.32	0.63	0.74	0.69	1.21	0.93	1.07
56	10.91	.	.	0.30	.	.	1.56	0.92	1.24
57	8.31	8.55	8.43	0.84	.	.	0.47	0.47	0.47
58	9.81	9.94	9.88	0.50	0.42	0.46	0.58	0.57	0.58
59	9.56	9.01	9.28	0.73	0.67	0.70	0.53	0.46	0.50
60	9.18	8.59	8.89	0.99	0.92	0.96	2.31	0.53	1.42
61	12.08	10.48	11.28	0.20	0.13	0.17	1.96	0.78	1.37
62	7.53	7.73	7.63	0.24	0.20	0.22	3.24	1.01	2.13
63	7.47	7.81	7.64	0.60	0.54	0.57	1.31	0.16	0.73
64	11.68	11.79	11.74	0.37	0.31	0.34	0.37	0.10	0.24
65	8.88	8.92	8.90	0.45	0.41	0.43	0.65	0.36	0.51
66	7.12	8.65	7.88	0.46	0.37	0.41	0.45	0.24	0.34
67	10.01	9.58	9.80	0.44	0.39	0.42	0.51	0.54	0.52
68	11.21	10.89	11.05	0.51	0.43	0.47	2.72	1.35	2.04
69	8.95	8.19	8.57	0.96	0.87	0.91	0.69	0.33	0.51
70	10.25	10.18	10.21	0.54	0.66	0.60	0.97	0.67	0.82
71	9.97	9.17	9.57	0.50	0.54	0.52	0.60	0.31	0.45
72	9.46	9.06	9.26	0.24	0.34	0.29	0.46	0.32	0.39
73	8.01	8.66	8.34	0.28	0.36	0.32	0.59	0.31	0.45
74	8.44	8.79	8.61	0.87	0.95	0.91	0.31	0.20	0.25

75	11.29	11.70	11.50	0.68	0.62	0.65	0.32	1.12	0.72
76	9.25	8.66	8.96	0.70	0.79	0.75	0.38	1.20	0.79
77	10.12	11.13	10.63	0.66	0.76	0.71	0.28	0.20	0.24
78	9.46	7.90	8.68	0.57	0.54	0.56	0.20	0.12	0.16
79	11.32	11.09	11.21	0.21	0.27	0.24	0.48	0.69	0.58
80	9.84	9.73	9.78	0.40	0.46	0.43	0.59	0.43	0.51
81	5.96	5.91	5.93	0.32	0.40	0.36	0.17	.	.
82	5.39	5.18	5.29	0.47	0.53	0.50	0.18	0.23	0.20
83	8.97	8.65	8.81	0.47	0.55	0.51	0.20	0.26	0.23
84	6.70	6.52	6.61	0.75	0.79	0.77	0.38	0.06	0.22
85	6.49	6.80	6.65	0.64	0.77	0.71	0.59	0.14	0.37
86	8.17	7.78	7.97	0.73	0.79	0.76	0.21	0.24	0.22
87	9.39	9.07	9.23	0.59	0.65	0.62	0.26	0.24	0.25
88	4.37	4.09	4.23	0.50	0.55	0.52	0.37	0.79	0.58
89	6.48	5.61	6.05	0.56	0.59	0.57	0.08	0.07	0.07
90	6.72	6.48	6.60	0.71	0.78	0.75	0.23	0.62	0.43
91	9.77	9.73	9.75	0.58	0.66	0.62	0.08	0.75	0.42
92	7.29	7.13	7.21	0.47	0.63	0.55	0.26	0.31	0.28
93	8.63	8.49	8.56	0.59	0.68	0.64	0.17	0.27	0.22
94	8.20	8.00	8.10	0.56	0.65	0.61	0.34	0.45	0.40
95	8.68	9.09	8.89	0.60	0.66	0.63	0.17	0.66	0.42
96	8.45	8.80	8.62	1.26	1.45	1.35	0.16	0.28	0.22
97	8.64	7.69	8.16	0.70	0.77	0.74	0.08	0.33	0.21
98	10.77	10.08	10.42	0.96	1.03	0.99	0.18	0.18	0.18
99	7.17	7.13	7.15	0.94	1.05	1.00	0.11	0.15	0.13
100	7.67	7.21	7.44	0.50	0.59	0.55	0.10	0.18	0.14
101	6.46	6.63	6.54	0.75	0.83	0.79	0.50	0.69	0.59
102	6.55	6.62	6.58	0.70	0.80	0.75	0.41	0.17	0.29
103	9.61	9.42	9.52	0.60	0.68	0.64	0.26	0.20	0.23
104	10.08	10.71	10.39	0.69	0.77	0.73	0.47	0.42	0.44
105	10.05	10.73	10.39	0.59	0.68	0.63	0.32	0.28	0.30
106	10.31	10.91	10.61	0.63	0.71	0.67	0.34	0.50	0.42
107	8.10	7.39	7.75	0.60	0.67	0.64	0.20	0.68	0.44
108	4.78	4.30	4.54	0.44	0.51	0.47	0.18	0.89	0.54
109	9.60	9.91	9.76	0.31	0.38	0.35	0.12	0.31	0.21
110	8.24	8.13	8.18	0.48	0.54	0.51	0.19	0.50	0.35
111	6.64	6.69	6.66	0.36	0.45	0.41	0.38	1.11	0.74
112	9.86	9.04	9.45	0.97	1.05	1.01	0.42	0.12	0.27
113	8.88	8.90	8.89	0.58	0.66	0.62	0.37	0.25	0.31
114	11.50	10.99	11.24	0.76	0.82	0.79	0.21	0.51	0.36

115	9.10	8.61	8.85	0.70	0.72	0.71	0.40	0.48	0.44
116	10.62	10.11	10.36	0.72	0.79	0.76	0.17	0.41	0.29
117	7.49	7.84	7.66	0.95	1.11	1.03	0.33	0.54	0.44
118	6.81	6.42	6.61	0.71	0.75	0.73	0.37	0.48	0.43
119	11.14	11.12	11.13	0.34	0.39	0.37	0.31	0.20	0.25
120	9.00	8.98	8.99	0.28	0.34	0.31	0.45	0.22	0.33
121	8.17	8.18	8.17	0.95	0.99	0.97	0.16	0.28	0.22
122	6.66	6.54	6.60	0.61	0.67	0.64	0.22	0.24	0.23
123	10.17	10.18	10.18	0.93	1.16	1.04	0.23	0.76	0.49
124	10.64	9.58	10.11	0.43	0.55	0.49	0.37	0.74	0.55
125	9.79	8.99	9.39	0.72	0.67	0.69	0.31	0.30	0.31
126	8.51	8.11	8.31	0.65	0.59	0.62	0.20	0.32	0.26
127	9.22	9.66	9.44	0.45	0.51	0.48	0.40	0.43	0.42
128	10.26	10.61	10.43	0.58	0.59	0.59	0.18	0.71	0.44
129	7.01	7.52	7.26	0.26	0.32	0.29	0.26	0.32	0.29
130	10.05	10.69	10.37	0.43	0.50	0.46	0.37	0.54	0.45
131	7.85	7.96	7.90	0.57	0.63	0.60	0.32	0.25	0.28
132	12.34	12.30	12.32	0.51	0.47	0.49	0.79	0.35	0.57
133	11.14	10.77	10.95	0.45	0.43	0.44	0.12	0.10	0.11
134	11.21	11.63	11.42	0.69	0.68	0.68	2.95	2.14	2.54
135	9.45	8.97	9.21	0.64	0.61	0.63	1.60	0.95	1.27
136	11.09	11.19	11.14	0.57	0.56	0.56	0.84	0.69	0.76
137	11.39	11.18	11.28	0.95	0.93	0.94	0.81	0.78	0.79
138	8.88	9.09	8.98	0.87	0.82	0.85	0.78	0.40	0.59
139	13.02	13.20	13.11	0.46	0.42	0.44	0.54	0.52	0.53
140	12.24	12.10	12.17	0.58	0.54	0.56	0.45	0.43	0.44
141	10.56	8.98	9.77	0.52	0.49	0.51	0.64	0.45	0.55
142	13.29	13.27	13.28	0.64	0.65	0.65	0.21	0.63	0.42
143	12.92	13.60	13.26	0.65	0.67	0.66	0.75	0.68	0.72
144	13.05	12.70	12.88	0.54	0.46	0.50	0.17	0.01	0.09
145	13.62	14.12	13.87	0.87	0.79	0.83	1.08	0.71	0.90
146	11.40	12.51	11.95	0.79	0.80	0.80	0.89	0.40	0.65
147	15.03	14.61	14.82	0.72	0.72	0.72	1.19	1.05	1.12
148	11.42	10.77	11.10	0.72	0.89	0.81	0.89	0.11	0.50
149	13.62	13.99	13.81	0.71	0.80	0.76	1.18	0.89	1.04
150	11.70	11.08	11.39	0.70	0.79	0.74	1.13	0.91	1.02
151	13.71	12.71	13.21	0.62	0.69	0.66	1.32	1.00	1.16
152	.	13.78	.	0.72	0.80	0.76	1.24	0.82	1.03
153	13.60	12.65	13.12	0.71	0.79	0.75	1.09	0.97	1.03
154	13.80	12.00	12.90	0.68	0.78	0.73	2.22	1.75	1.98

155	13.66	13.03	13.34	0.87	0.96	0.91	1.33	1.02	1.17
156	14.60	14.59	14.60	0.87	0.92	0.90	1.51	0.72	1.12
157	13.89	12.31	13.10	0.46	0.53	0.49	1.27	1.68	1.48
158	12.57	14.18	13.37	1.49	1.61	1.55	1.17	1.27	1.22
159	13.70	13.61	13.65	1.94	2.12	2.03	0.83	1.05	0.94
160	13.18	13.60	13.39	0.87	0.85	0.86	0.89	0.92	0.91
161	15.18	12.70	13.94	0.71	0.36	0.54	0.97	0.62	0.79
162	13.44	12.58	13.01	1.02	1.03	1.03	1.10	0.44	0.77
163	14.32	15.70	15.01	1.09	1.40	1.25	0.90	0.17	0.53
164	11.87	10.98	11.43	0.85	0.91	0.88	0.95	0.22	0.59
165	15.75	15.07	15.41	0.76	0.79	0.78	1.35	0.05	0.70
166	16.58	14.18	15.38	0.96	1.13	1.04	0.97	0.31	0.64
167	11.09	11.09	11.09	1.09	1.41	1.25	1.20	0.59	0.90
168	.	11.88	.	1.01	1.03	1.02	1.11	0.14	0.62
169	15.01	15.16	15.09	1.11	1.31	1.21	0.84	0.13	0.48
170	13.83	14.07	13.95	0.96	1.10	1.03	0.91	0.15	0.53
171	14.05	14.08	14.06	0.99	1.17	1.08	0.83	0.38	0.60
172	14.50	13.81	14.16	0.95	0.96	0.95	1.20	0.76	0.98
173	13.21	12.57	12.89	1.02	1.38	1.20	1.21	0.80	1.00
174	13.77	13.09	13.43	1.19	1.03	1.11	1.22	0.70	0.96
175	13.07	13.30	13.18	0.94	1.08	1.01	0.74	0.92	0.83
176	14.19	14.00	14.10	1.04	1.03	1.03	1.09	1.11	1.10
177	11.25	11.74	11.49	1.26	1.39	1.33	0.76	0.33	0.55
178	13.00	12.20	12.60	0.98	0.89	0.94	1.01	0.34	0.67
179	13.35	11.61	12.48	1.22	1.29	1.26	1.25	0.62	0.93
180	11.22	10.85	11.03	0.95	0.95	0.95	1.29	0.58	0.93
181	12.32	12.60	12.46	1.11	1.46	1.29	1.13	0.79	0.96
182	13.03	12.18	12.61	1.20	1.08	1.14	1.02	0.63	0.82
183	14.89	14.30	14.60	1.34	1.38	1.36	1.12	0.68	0.90
184	13.43	12.52	12.97	1.41	1.50	1.45	0.37	0.49	0.43
Parent1	15.51	15.84	15.68	1.44	1.51	1.48	1.04	1.40	1.22
Parent2	11.33	11.91	11.62	1.04	1.14	1.09	0.87	0.70	0.78
