# Supplementary material for: QTL mapping of selenium content using a RIL population in wheat
Source: PLoS One. 2017 Sep 7;12(9):e0184351. doi: 10.1371/journal.pone.0184351 (PMC5589217; doi:10.1371/journal.pone.0184351)
Supplement: S2 Table — (PDF) [file pone.0184351.s002.pdf]

S2 Table. Basic information of the four markers related with *QSsec-4B* on 4B chromosome

Marker name	Chromosome	postion	Type of the markers
wPt-744595	4B	0	DArT (Wheat PstI (TaqI) V3)
wPt-7233	4B	1.817	DArT (Wheat PstI (TaqI) V3)
wPt-8555	4B	3.139	DArT (Wheat PstI (TaqI) V3)
Jagger_c10704_106	4B	5.169	SNP 90K
