# Supplementary material for: QTL mapping of selenium content using a RIL population in wheat
Source: PLoS One. 2017 Sep 7;12(9):e0184351. doi: 10.1371/journal.pone.0184351 (PMC5589217; doi:10.1371/journal.pone.0184351)
Supplement: S3 Table — (PDF) [file pone.0184351.s003.pdf]

S3 Table. Sequence of the four markers related with *QSsec-4B* on 4B chromosome

Marker	Sequence
wPt-744595	tgcagagcaaaaacctggacagatctcccttgctctctgggaagtagctacaggtttcagtggcttgagctggtactgaacaaaaagtcatctgtatttctacctttatcacacatcaacaattccaagcttaaaattggttgatgcacgtcgccgccatggaaaagaagg<br>ataattagattttagtcactagagacaatcaacaactccttctaaagggaaatgtggcttgcacacaaagttgaggagacaaataattttagagagtaaagaatataagtaaagtagtgtggtggtctggtggagcaggaatcagttaggtacctagatgagagacatat<br>ctgaatgtccgcgagcatgttggtgatataggttgctaaagcatcaagaaccgctgcccctggttctgggttccttctcccttgagctggtactgggaacaagcatgcaaataaaaattagtcctcgtcattagtacctatcacacatcaacaatcccatgctaaaatctga<br>ttgatgtgaaataagattcactcactgca
wPt-7233	tgcagatcaatgtttgtctccatgctgttggtttcacttgtgtaaaataggtaccatttcttttcaatatcttcataaagatcatctgcataagcctcaacgtgcaggatatgattgaacttgtcccactcggggccatattttcccaaagccatggaagcgagtacttttgggca<br>gcagtccagcaacaaatgacttgactttacagtttgtaggtacatcgggagttgtccctcgtcatagtgaactgtcagcacgagtctctggagtgcatttatctcctgca
wPt-8555	tgcagtacctcttcagaacccaaactctccccttggtcccactgtgcacgcgggcagtaaggcatttctgcttctcgctcatccaccttggacaccctcggtttctcaacctcgtctaggaatttcctgatgatatccaagttggagttcttgagcacccatactctctcccc<br>atacttgagtacaccagccacaaatatcagcatggcggttgtggccaaggtccagctgtcagcgacatacttgtaaaggacataagctactccaagggtctgcaccacaagagagagcaggtggcgtggccagagctgttgtcttccatggcggtgatggtgtcct<br>ggccaccgaggtgcaccagcaggaatggcgcccagaatgccaccagctgca
Jagger_c10704_106	acaacaacagactctagccgaaaaagaacatgcatatgtgggtggcaatg[t/c]tgtgctgcaaacaaataaagttgatggtgaatctagttgctcagctgttg
