# Supplementary material for: QTL mapping of selenium content using a RIL population in wheat
Source: PLoS One. 2017 Sep 7;12(9):e0184351. doi: 10.1371/journal.pone.0184351 (PMC5589217; doi:10.1371/journal.pone.0184351)
Supplement: S4 Table — (PDF) [file pone.0184351.s004.pdf]

S4 Table. Significant alignments of the four markers related with *QSsec-4B* on 4B chromosome

Marker	Sequences producing significant alignments
wPt-7233	TGACv1_scaffold_328822_4BS
wPt-744595	TGACv1_scaffold_337086_4BS
wPt-8555	TGACv1_scaffold_328580_4BS
Jagger_c10704_106	TGACv1_scaffold_332206_4BS

Note: sequence was blast on line: http://www.sequenceserver.com/
